# Throat-Ail

**Published:** 1881-11

**Authors:** 


### THROAT-AIL.

I have endeavored in all my writings to substitute this name for *Laryngitis*, or *Clergyman's Sore Throat;* it is shorter, more comprehensive, more correct, and has the advantage of being plain English. It is a disease which every mother ought to understand, for in the shape of *croup*, it puts her child in the grave in a few hours. Every person who loves to sing, should know its nature, for it destroys the voice. Every lawyer, every clergyman, every politician ought to make it their study, for it robs them of their capital in trade, and often lays them on the shelf for life. In short, it should be generally understood at least as to its symptoms, for it is very often the forerunner of consumption, that hated name.

There are two forms of throat-ail—the rapid and the slow. By rapid throat-ail, the great and good Washington perished prematurely, in a few hours' illness. By the slow kind, many public men are deprived of their means of usefulness, and of support, and have to spend their remaining days in struggling for a scant subsistence, or in following some new trade in their old age.

I write for the people, and think it sufficient for the general good, to acquaint my readers with merely the symptoms and the causes of what is called "THROAT-AIL," par excellence, the kind which lasts for weeks and months, and years, ending in disablement of voice, and finally death by consumption.

*Throat-Ail* is like a fire, the sooner you know of its existence the better; and like a fire too which seldom goes out of itself; so throat-ail seldom indeed gets well of itself, but burrows and deepens, until it undermines the constitution, wastes away the health, and strength, and flesh, and finally fastening itself in the lungs, completes the wreck and ruin of the whole man.

The first symptoms of Throat-Ail, or Chronic Laryngitis, or Clergymen's Sore Throat, are usually a frequent hemming and hacking, in order to clear the voice or throat; this is slight and seldom at first, and may not be noticed for weeks; but then, it is so decided, that it forces itself upon the attention, either by its frequency, or by the force required to clear the throat sufficiently to speak with distinctness. After a while, it requires such an effort to enunciate plainly, that the patient for the first time becomes aware of a certain feeling of tiredness about the throat or neck; most generally it is a dull hurting; or he finds there is a kind of lumpish feeling in the throat, and he attempts to swallow it away, and it does seem to go down, but it does not stay down, and he swallows again, and soon he finds himself *swallowing all the time;* occasionally there is a different cause for swallowing, the throat appears to be dry, and swallowing for a time seems to moisten it; finally the swallowing is almost incessant, especially if the mind is directed to it. For a time, nothing is brought away; gradually a little pearly or whitish or cottony like phlegm is brought up, and the patient becomes hoarse. In the progress of things this phlegm becomes dryish, and so tough, that it clings to the inside of the throat, and can only be dislodged by a decided effort at clearing, with a dipping forward of the head. The voice next becomes husky; at last a positive cough is necessary to dislodge the phlegm, and consumption soon follows.

The symptoms detailed are present in the history of every case I have known. Accompanying these, there are occasional additional symptoms. A kind of pain, sharp or hurting, runs up the side of the neck towards the ear. Some complain of a burning feeling now and then at the little hollow at the bottom of the neck; or up and down the breast bone in the centre, or at the pit of the stomach. These burning sensations are not felt continuously in any case, but at certain times during the day.

A very common symptom is a depression of spirits, altogether greater than the actual feeling of discomfort warrants. In the progress of the disease, the feet become cold; there is a bad taste in the mouth of mornings; occasional headache; the bowels do not act daily, or if they do, what is passed is hard or bally; the patient is easily chilled; "the slightest thing in the world" gives him a cold, and "a cold always makes the throat worse." The food either sours on the stomach, or remains there like a weight for hours at a time; the appetite becomes impaired, or it is so voracious, that "I can eat almost any thing" and "yet hungry all the time." The patient begins to lose flesh and strength; and does not swallow as easily as he used to; at length he cannot swallow at all; in the effort, even water comes back through the nose and the man dies of starvation.

Reader, if you have incipient symptoms of throat-ail, do not be a fool and go to some old woman, or Indian Doctor, or some officious and all-knowing granny, and waste time and perhaps life in experimenting on red pepper tea, or the soup made by Shakespeare's witches, or the Alicumstouton Salve, named at page 147 of the Journal for 1854. Do not go to swallowing brandy, or the still more murderous lozenges of the shops; for brandy may not certainly kill any man, lozenges will. But go at once to a regularly educated physician, who is, as I think, necessarily a gentleman; he will not promise to cure you in a week, or in a month, or in a century; he will promise you just nothing at all; he takes it for granted that you understand that he feels it his duty and his interest to do for you the best he can, and he will do it. Do not tell him that if he cures you, there are a few more of the same sort left in your neighborhood who will also come. Do not promise him an extra fee if he is successful in your case; for it will only make him feel that you are as green as you suppose him to be. Do not come the pathetic over him, that you have six wives living and dead, and nineteen children, and you hope he will do the best he can for you, for the—smallest price possible. In calling upon such a physician, you have only two things to do; tell your symptoms, and follow his advice implicitly and well; his reputation and his bread depend on his success: you can appeal to no higher motives. And always remember, that it is impossible for such a physician to say to you, "no cure no pay." Is a man to spend weary hours and anxious days and sleepless nights in trying to save your life, and to be paid nothing, unless he succeeds, especially when you have spent all your money on patent medicines and advertising certifyers; shame on the man who could make such a proposition.

### CAUSES OF THROAT-AIL.

I cannot here state them all, nor at length, only the principle ones, and them succinctly.

I have now these many years confined my attention rigidly and exclusively to throat and lung diseases. I think I was the first physician in the United States to do so, as rigidly. I know not that there is any one besides myself in this country, who dismisses every case as invariably, in which the air passages are not involved. I make this statement for the purpose of enabling the reader to place the deserved estimate at the assertion I am going to make, to wit:

*Three cases out of every four coming to me for throat-ail, have it as the result of improper eating and drinking.*

Such a large proportion of cases of throat-ail originating in the stomach, I found my remaining remarks on this general origin.

*How can the Stomach make the Throat Sore?*

A stroke against the elbow is felt at the fingers' end. When your foot is asleep, from sitting on a hard edge of wood for some time, the cause is at the point of pressure, and yet it tingles in the toes a yard off. A good knock on the head "makes the fire fly" at the eyes.

The condition of the throat is affected by the condition of the stomach, because a certain nerve branches off, one part of that nerve goes to the stomach, the other fork goes to the throat. The nerves are like the telegraphic wires, touch them at one end, and an effect is produced at the other. So if the nerves which supply the stomach are disordered, those in the throat are liable to become so too. Most of us have heard of "*heartburn*," some have felt it; it is a burning sensation, sometimes felt at the point familiarly called *the pit of the stomach*, and sometimes in persons who use their voice much, this same burning is felt at the little hollow at the bottom of the throat and the region of *Adam's apple*, and that is the spot where throat-ail is located.

I wish here to arrest the attention of clergymen, singers, teachers, and public speakers to this interesting inquiry.

If sour stomach, or *dyspepsia*, as physicians term it, causes burning or other sensations in the throat of clergymen and other persons who use their voice much, why does not sour stomach affect the throats of all, as the same nerve supplies branches to both throat and stomach? This is the reason: a slight stomach derangement does not affect the throat perceptibly, if the voice organs are in a strong, active, healthful condition, because they have vigor to repel disease. It is a law of the human frame, that an ailment is apt to make itself felt next, or most decidedly in that particular part of the body which at the time is weakest in the performance of its functions, and as the voice organs are often in a lax or debilitated condition from frequent or unusual voice efforts, or injudicious conduct after voice effort, as stated at length in the Journal for 1854, page 39, and are at length made permanently feeble by these repeated uses and indiscretions, so being the next weakest part, disease flies there; thus it is too, that when such persons take cold, the throat being the weak part, feels it promptly.

A proper use of the voice strengthens the throat, and gives it a capability of resisting disease, just as a judicious use of any other muscles of the body increase their strength and health. But improper use, as just stated, by weakening, renders them more susceptible of disease of any kind, and specially of the stomach, in consequence of the nervous connection before described.

An injury done to any part of the body may be resisted, or if not, may be repaired by the curative energies of nature; but if these injuries are frequently repeated, the strength of nature is exhausted in endeavoring to make repairs, then she remains prostrate and powerless, and disease has unbridled sway.

When in any given case, a man is in a condition to have his throat affected by the state of his stomach, violence is offered the throat at each meal, three times a day, in time these effects last longer, until the effect of one meal reaches to another, and the throat is more or less ailing all the time.

But to follow up the case, how is it that persons have *sour stomach* or *heartburn?*

All understand that what is sweet cider to-day, is sour tomorrow; we look at it and find it in constant motion, it is "*working*," *fermenting*. When food is taken into a healthy and well acting stomach, it is in a short time *digested*, that is, converted into a kind of liquid, no lumps or any thing of the sort in it, just as when you place a great many bits of ice and snow in a glass of water, the mass soon becomes all fluid alike. The food is made into this one fluid substance by the action of the stomach and what pertains to it. But the amount of food which the stomach can thus turn into a liquid form, is limited, just as if you put a certain amount of ice lumps in a glass of water, that water will melt them, but if you put in too many, none of them are wholly melted, and it remains a mixture of water, spears of ice, and solid ice. When then, more food is taken into the stomach at any one time than it can convert into a homogenous fluid, it remains in lumps more or less, and it is said to be *undigested*, and begins immediately to ferment, to become sour and produces in the stomach the same sensation that swallowing vinegar causes in the throat, a burning.

We see then, that sour stomach is caused by eating more than the stomach can digest. But how are we to tell how much the stomach can digest? In the same manner precisely as each one may ascertain to a quarter of an hour how much sleep he needs as explained in the Journal for 1854, page 88. Observe nature. The brutes are regulated in all these things by instinct, to us the nobler reason is given, and it must be our guide. We must observe and judge.

What one man eats or drinks in quality or quantity is no guide for any other man, any more than the amount of labor one can perform, is the criterion for another. Each man must for himself bring his own observation and judgment to bear on the question, *How much must I eat?* The general rule is, Do not eat so much, as to cause any unpleasant sensation after wards.

If you at any time take a meal, and afterwards within an hour or two feel uncomfortably, then what you have eaten, *does not agree with you;* you have eaten, either in quantity or quality what your stomach cannot digest. Nine times out of ten, it is the quantity and not the quality, which does the mischief.

When persons have been ailing some time, almost every thing they eat or drink, sours on the stomach, even a cup of tea or a glass of cold water, or toasted bread, gives sourness, or weight, or oppression, or some other ill feeling; in time, the throat begins to feel tired, dry, or to burn, or smart, or is clogged up a little and we are all the time clearing it away; this is "*Dyspeptic Throat-Ail*," or Clergymen's Sore Throat. But why was such a name given to it? Because to a certain extent it is a comparatively new disease, we read little or nothing of it in the old books, a new disease as much then, as cholera is a new disease. It was perhaps first noticed to attack clergymen for two reasons: the injudicious use of the voice, as noticed in the article on Air and Exercise for February, 1854; and from increased notoriety over a common patient, for when the minister is ailing the whole town and adjoining country soon know it; but I am now come to the point of exposing one of the two grand mistakes of modern times in reference to health. I will name them both here, although I will at present discuss but one. The first mistake is about injuring one's health by hard study, and the other is that a minister has become disabled by his "*arduous labors*;" these two things are simply pious frauds, the former committed generally by young students, the latter by young clergymen, securing for them a kind of sympathy considered to belong to martyrs. Two things I know: the first is, I never injured my health by hard study; the nearest I came to it was in ruining my eyes by studying the miserable edition of Scrivilleis' Lexicon, "a long time ago," till twelve o'clock at night, the days having been spent in writing poetry and pathetic epistles to a schoolmate. I received sympathy instead of the switch, just as nine young gentlemen out of ten in the college, the university, and the lecture room are complimented, when their health gives way, with the appellation of a *hard student*. I never knew a man, young or old, to injure himself by hard study. It is a mistake. In some future number I may tell how said mistake originates.

The other of the two grand mistakes before alluded to, I propose to discuss is this, "*Clergymen's sore throat is wrongfully set down to the score of* '*arduous labors!*'" Let the observant reader reflect a moment on a little fact which ravenous appetite, that an amount is eaten very far beyond the needs of the system, keeping the stomach laboring for hours after, to relieve itself of the unwonted burden. Such occurrences frequently taking place, will inevitably induce dyspeptic habits, and all their long catalogues of ill. *Our ministers are feasted too much.*

Another cause of dyspepsia in ministers, is *eating too soon after preaching.* For two or three hours the tide of nervous energy has been setting in strongly towards the brain, and it cannot be suddenly turned towards the stomach; but the mental effort has occasioned a feeling of faintness or debility about the stomach, and a *morbid* appetite; and if food is taken at all largely, there is not the nervous energy there requisite to effect its digestion ☞ for the brain will be running over the discourse; you may bring the mind back to the eating for a moment, but before you are aware of it, it will be laboring at the discourse again; every public speaker knows this, and the food lies there like a weight or a lump for hours.

The same result is produced in a less decided form by studying out a sermon. The mind becomes absorbed, the announcement for dinner is made, you are unprepared for it, it is rather unwelcome, you do not feel hungry, for the brain is at work, not the stomach; however, as it is meal time, you go down, but the mind is in your "study," and you eat because it is dinner time, and not because you have an appetite—the principal cause of the most aggravated forms of dyspeptic disease—*eating without an appetite*, one of the most suicidal of all domestic practices; eating simply because it is eating time, rather than by waiting until the appetite comes, give the trouble to prepare another meal. Every student should leave his books at least half an hour before a meal, and spend that half hour in a leisure walk in the open air, or in agreeable conversation on the piazza, or in the garden.

### AN INSTRUCTIVE WARNING TO CLERGYMEN.

In illustration of the principles stated, I will record here a fact.

A very eminent D.D. *within a year* has given up the charge of his congregation from *a complaint in the throat:* his parishioners, in parting with him, presented him with a farm, and now he is lecturing over the country, and nothing is heard about his throat complaint, except when he leaves his wife at *home;* when that is the case, he is laid up instanter As long as she is at his side to watch over what he eats as to quality and amount, he keeps well; when he transgresses, the food sours on the stomach, the throat burns, gets clogged up, he is hoarse and useless.

I have extended this article beyond my calculation, but its importance cannot be over estimated, for I consider it a statistical fact, that *three out of four of all the clergy who are prematurely set aside as unavailable workers*, are thus set aside in consequence of errors in diet; errors to a certain extent inseparable from their present connection with society, in the manner I have stated.

Throat-ail then being generally located in the stomach—what is the use of gargling the throat with acids and metallic preparations, which destroy the teeth? and what is the use of swabbing out the throat with nitrate of silver, when the source of the disease is elsewhere. It does I know sometimes give relief, but it is not permanent, it cannot be, for it is merely covering a black spot on the wall with whitewash; the spot is not seen, but it is there still; but unlike the black spot, which is statu quo, the disease, though covered, is burrowing and spreading still. If again, the disease is really in the stomach, it is a useless waste of time, it is unphilosophical, to tell a clergyman who has throat-ail, that he must abandon preaching; because the voice muscles must be treated like any other muscle of the body which is debilitated, their energies must be invited back by judicious forms of exercise, just as in recovering from a fever, we increase our strength, by exercising carefully and gradually, and safely increasing that exercise.

Besides, if the minister gives up his congregation, he gives up his bread, and he not only has leisure to brood over and thus aggravate his ailment, but also to worry himself as to some mode of obtaining subsistence in a manner not inconsistent with his former calling. Hence, the indispensable means of curing an ordinary case of clergymen's sore throat are to keep the patient at work, modifying the forms of voice exercise according to the needs and habits of each case, and the regulation of the digestive functions by a proper adaptation of food as to quantity and quality to the needs of the system.

### COLD FEET

**Often** produce a burning sensation in the throat, which if allowed to continue in operation, ultimately undermines the health; the reason is, less blood being in the feet than is natural, there is an extra amount at the other end of the body; can any thing be more absurd than to clip off a man's palate, whack out his tonsils, and "burn out his throat," for such an ailment; can that send warmth to the feet? can we purify the fountain, by purifying the stream? When will men learn to think for themselves?

My experience is, *Throat-Ail is not to be radically and permanently cured in any case, except by rectifying first, and then building up the general health of the system*, and that requires time, determination, and systematic habits of rational life. Who thinks differently, and acts up to his belief, will find himself just as miserably deceived, as that unfortunate class of theologians, who assert "It is no matter what a man believes, if he is sincere in his belief." Is not such a logician a "sincere" fool? *Clergyman's Sore Throat is better cured*, AS A GENERAL RULE, *in the continuation of ministerial duty.* My ordinary advice is, PREACH EVERY DAY AND SUNDAY TOO, rather than once a week. These fitful efforts are often a main cause of Throat-Ail; just as a man who travels ten miles a foot on Sunday, and on other days none at all, will be wearied every Sunday night; whereas, were he to walk five or six or eight miles every day, rain or shine, he would perform ten or twelve on the Sabbath, without appreciable fatigue. Men of "The Cloth," why don't you think for yourselves? Sometimes I think I am not altogether a drone in creation, because there are excellent men now, in different parts of the country, whom I have never seen, who, having abandoned preaching, applied to me for advice, and on being urged to resume pastoral charges immediately, as a means of cure, have done so, and have steadily recovered, and are now bearing "the burden and heat of the day." So that I am every Sabbath preaching by proxy, to many a listening multitude. It is not politic to say here how many I have killed off, or to inquire if those referred to might not have recovered without doing any thing. They came and were cured as antecedent and sequent, not necessarily as cause and effect.

